# Collagenous colitis in a child induced by chronic respiratory allergy: A case report

**DOI:** 10.1097/MD.0000000000021920

**Published:** 2020-08-28

**Authors:** Xue-Meng Wan, Zhi-Ling Wang, Li-Yuan Wang, Xiao-Tang Cai, Chao-Min Wan, Yong-Mei Xie

**Affiliations:** a Department of Pediatrics, Sichuan University West China Second University Hospital, NO. 20, Section 3, Ren Min Nan Lu Road; b Key Laboratory of Birth Defects and Related Diseases of Women and Children (Sichuan University), Ministry of Education, Chengdu, Sichuan, P.R. China.

**Keywords:** allergy, collagenous colitis, pediatrics, respiratory tract

## Abstract

**Introduction:**

Collagen colitis (CC) is a microscopic colitis diagnosed by mucosal biopsy and is extremely rare in children.

**Patient concerns:**

We reported a child with severe persistent diarrhea that could not be relieved with traditional diarrheal treatment. No abnormalities were found after multiple colonoscopies.

**Diagnoses:**

A significant increase in total IgE levels was found in the patient's blood. He had a history of mild chronic allergic rhinitis and slightly intermittent wheezing. However, we found that the child had a hyperallergic reaction to multiple respiratory antigens and had mild pulmonary dysfunction. Finally, colonoscopy with biopsy identified the diagnosis of CC.

**Intervention:**

Considering that a respiratory allergic reaction was one of the causes of diarrhea, anti-allergic treatment was given to the child, and his severe diarrhea was soon relieved. Corticosteroid treatment was suggested to the patient, but his parents firmly refused steroid therapy. According to the patient's specific allergic reaction to mites, desensitization treatment was finally chosen for him.

**Outcomes:**

After 1 year of desensitization for dust mites, the patient's respiratory symptoms improved, total IgE levels decreased, autoantibodies declined, and diarrhea did not reoccur. Colonoscopy with biopsy showed a significant improvement in pathology.

**Conclusion:**

CC in children is rare, and childhood CC induced by a respiratory allergic reaction has not been previously reported. Therefore, this is a special case of CC in a patient who was cured with anti-allergy treatments and desensitization instead of steroid therapy.

## 1 Introduction

Collagen colitis (CC) is a type of microscopic colitis (MC) with a morbidity of 49.21/100,000.^[1]^ It is mainly diagnosed by typical histological changes under mucosal biopsy. CC in childhood is extremely rare.^[2]^ The pathogenesis of CC had still not been fully elucidated, but the possible related factors may include immunity, allergy, genes, and drugs.^[3]^ A typical clinical manifestation of CC is chronic, non-bloody, watery diarrhea,^[4]^ that is self-healing or repeatedly occurs. CC can be intermittent or can persist for months or years.^[5]^ Diagnosis of CC depends on colonic mucosal biopsy.^[5–7]^ Treatment of CC aims to achieve clinical remission and improve patients’ quality of life.^[4,8]^ Moreover, the objective of curing CC is to find its etiologies and predisposing factors and treat the causes. Previous research regarding CC in children is extremely rare, and most cases have unclear causes. The child in this case had diarrhea symptoms similar to CC and was diagnosed with CC by colonoscopy with biopsy. Finally, chronic allergic reaction in the respiratory tract was identified as the pathogenesis of this case, which has not been reported before both in CC cases of children and adults.

## 2 Case presentation

### 2.1 History and physical examination

The patient was a 10-year-old boy of Han nationality. He was admitted to our hospital because of “intermittent fever and persistent diarrhea for 23 days.” Dietary inducing or infection-related causes were not found during the onset of diarrhea. He had serious diarrhea more than 30 times per day and passed stools about 10 times during the night. The stools were yellow and watery, had no special odor, and were without blood and pus. He passed 30 to 100 ml of watery stools each time, without urgency and anal discomfort. Mild abdominal pain often accompanied, which was mainly focused on the navel, and mild nausea or vomiting without abdominal distention was occasionally present. He had a repeatedly low fever, without shiver and febrile rash. Several routine examinations were completed in the primary hospital, and routine blood examinations, C-reactive protein, procalcitonin, thyroid function, stool cultures, and virus detections were normal. Multiple routine stool examinations showed positive occult blood without other abnormalities. No apparent abnormalities were found after two colonoscopies in the primary hospital. Oral rehydration salts, smecta, probiotics, racecadotril, nystatin, and other routine treatments were given to the child, but severe diarrhea and low fever were not relieved. Before being transferred to our hospital, the patient had a 2 kg weight drop, mild appetite loss, and poor sleep due to frequently passing stools. Dietary inducing factors and epidemic traveling history were not found. Moreover, he had two hospitalizations due to “wheezing pneumonia” in the past 6 months. No specific abnormality was found in his family history. Physical examination showed mild dehydration and slight malnutrition. Palpation of the abdomen was soft, with mild unfixed tenderness and pain. No tension of the abdominal muscles and no rebounding pain were present. The patient's liver, spleen, and anal region were normal, and bowel sounds were sometimes active. Other systems, such as the lymph nodes, heart, lungs, and nervous system, were all normal.

### 2.2 Routine clinical laboratory examination

Routine blood tests were mostly normal, and only a mild increase in the percentage of eosinophils was found, which was 7.9% (standard 0.5%–5%). Routine stool tests were all normal, and fecal flora examinations showed mild flora disorder. Fecal detections for virus antigens were all negative, including rotavirus, adenovirus, and norovirus. Several cultures taken for stool, blood, and bone marrow were all negative. The patient's bone marrow smear was normal, and no parasites or protozoa were found. Routine blood examinations for liver function, kidney function, thyroid function, electrolytes, and erythrocyte sedimentation rate were all normal. Clostridium difficile toxin A/B, hepatitis B, and human immunodeficiency virus antibodies were negative. The patient's tuberculosis T-cell spot test and two purified protein derivative skin tests were normal. Detections for fungal infection were normal, including the fungal G test, galactomannan test, and fecal fungus culture. Tests for typhoid, paratyphoid fever, and TORCH (toxoplasma, rubella virus, cytomegalovirus, herpessimplxvirus) were normal. Total serum IgG, IgM, and IgA levels were all normal; however, total IgE levels were significantly increased (1970.0–2100 IU/ml [standard <165 IU/ml]). Therefore, screenings for parasites were performed, such as the parasite-specific antibody, imageology, and repeated stool examination, all of which had no positive findings. The possibility of food allergy was considered. Food allergen-specific IgE was subsequently detected and showed negative results. The detection for food allergen-specific IgG showed milk (1+), soybean (1+), and corn (1+) as potential allergens, but this was too weak to diagnose a food allergy. Subsequently, respiratory allergen-specific IgE was sequentially detected, which showed house dust mite (3+), dermatophagoides farinae (3+), and house dust (3+) allergies. At the same time, detections of blood autoantibodies showed that antinuclear antibody (ANA) concentrations were 1:1000 (standard <1:100) and anti-histone antibody (AHA) concentrations were 347.9 RU/ml (standard <20 RU/ml). An enhanced computed tomography (CT) scan of the abdominal region showed no abnormalities except slight pelvic ascites.

### 2.3 Colonoscopy and pathological biopsy

**Figure 1:**
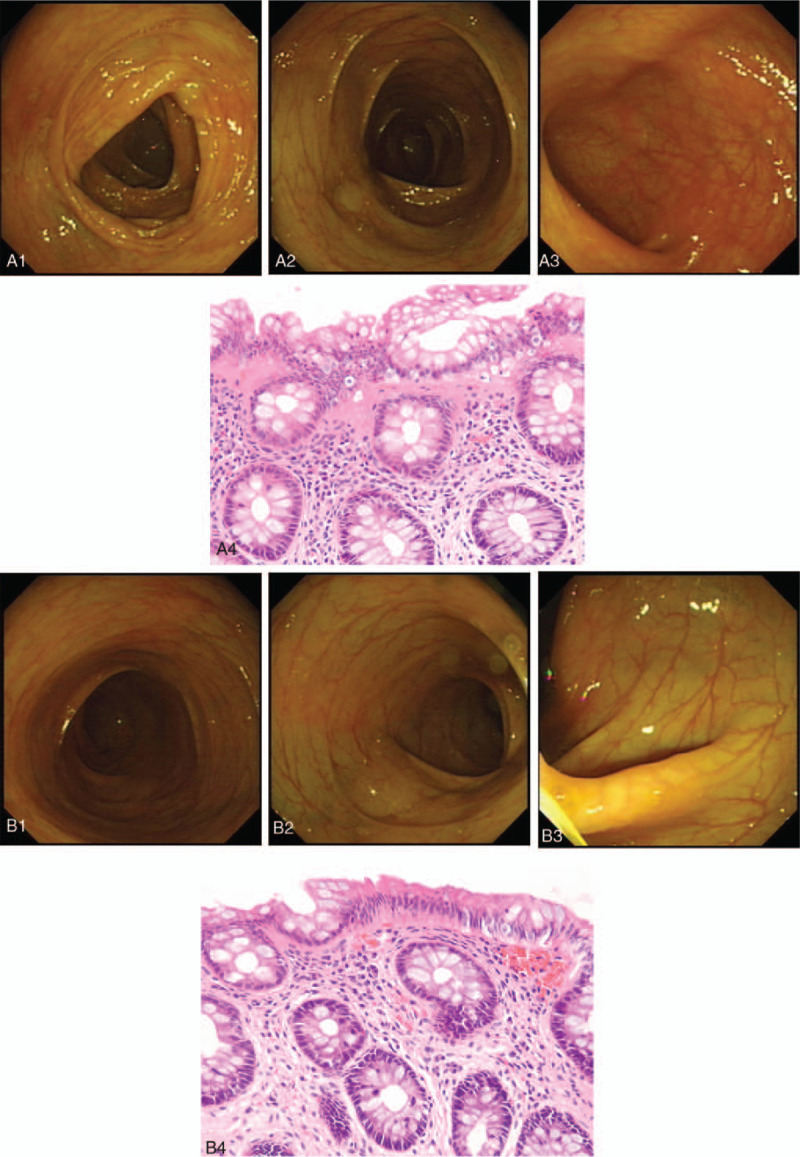
Colonoscopy and pathological biopsy.

The patient had undergone two colonoscopies in the primary hospital, but no significant abnormalities were found. Therefore, biopsies were not performed. During admission to our hospital, the patient's colonoscopy was re-checked on January 24, 2018. Only slight hyperemia was seen on the rectum and sigmoid colon, but the other parts of the colon were normal (see Fig. 1A1–A3). Biopsies of multiple sites were performed for the pathological examination. Since several rare signs were seen with microscopy, the pathology department made a general discussion for this patient, and finally got a definite pathological diagnosis. The pathological examination showed that extensive invasions of the lymphocytes and plasma cells were found from the terminal ileum to the rectum. Glandular atrophy was also present in several regions of the colon, and broad collagen bands were clearly seen under the mucosal epithelium in all biopsies of the colon samples. Therefore, the final pathological diagnosis was considered as “collagenous colitis” (see Fig. 1A4).

### 2.4 Treatment during hospitalization

After admission to our hospital, the patient had intermittent low fever and severe diarrhea more than 30+ times per day. Symptomatic treatment of intravenous rehydration, smectite powder, and racecadotril was given to the patient, and although this treatment helped relieve dehydration, it did not have a beneficial effect on his diarrhea. Since increases of total IgE and food-specific IgG were found, mebendazole was given for 3 days, and the elimination of suspected foods (eggs, milk, soybeans, corn) was suggested to the child. However, severe diarrhea was not alleviated and persisted for 2 weeks. Specific IgE of respiratory allergens was detected and showed a strong positive correlation to multiple respiratory allergens. According to this result, we carefully inquired about a history of the respiratory system again. A history of mild chronic allergic rhinitis and several episodes of wheezing pneumonia was found. His parents thought that mild respiratory symptoms had no relationship to diarrhea and never informed doctors of his respiratory history before. A chest CT scan was performed, and no abnormality was found. However, lung function tests suggested increasing resistance in the diffuse small airways. Therefore, anti-allergic treatment was given to the patient, considering a respiratory allergy. A combination of montelukast sodium, loratadine, and cyproheptadine was administered to the patient. After 2 days of anti-allergic treatment, severe diarrhea remarkably decreased from 30+ times/day to 10+ times/day. Five days after changing the therapy, the patient's diarrhea relieved, and his stool completely turned dry, and he began passing stools 1 to 2 times per day. At the same time, the child's temperature returned to normal, and his general condition significantly improved. The percentage of eosinophils in the peripheral blood also returned to normal; however, the patient's total IgE level was still considerably high (1790 IU/ml). At this time, the report of the pathological biopsy came out, and the final diagnosis was made clear as collagenous colitis (Fig. 1A4). According to the pathological diagnosis, corticosteroid treatment was suggested to the patient, yet steroid treatment was firmly refused by his parents because of the complete relief of diarrhea after anti-allergic therapy. The patient was discharged with continued oral use of montelukast sodium and loratadine and was required to follow-up in the outpatient department.

### 2.5 Follow-up in the outpatient department

At the 1-month follow-up post-discharge, the child was in good general condition with the maintenance of montelukast and loratadine, no recurrence of diarrhea, no abdominal pain, and no fever. However, his total IgE levels (1780 IU/ml) and autoantibody titer (ANA 1:640, AHA 150 RU/ml) were still high. Short-term corticosteroid treatment was suggested again and was refused again by his parents. Since most of the patient's respiratory allergens were dust mites, desensitization of dust mites might have been an alternative therapy to steroids. A consultation between the respiratory and gastroenterology departments was conducted for this patient. The respiratory department suggested that inhaled steroid therapy was unnecessary for the child because of no current respiratory symptoms. Desensitization of dust mites was also suggested to the patient by a respiratory specialist.

At the 3-month follow-up, the child had started desensitization treatment. The following steps were the detailed protocol for the desensitization therapy: solutions of dust mites dropped under the tongue with solutions Nos. 1, 2, 3 (protein titer, 1, 10, 100 μg/ml, respectively) during weeks 1, 2, and 3; No. 4 solution (protein titer 333 μg/ml) dropped under the tongue once a day after week 4 and subsequently for 2 years. Three months after discharge, the patient was in good condition without any gastrointestinal and respiratory symptoms. Although his total IgE (1028 IU/ml) and ANA (1:320) levels were still high, they began to decrease. AHA antibody levels turned negative. We suggested maintaining montelukast and desensitization treatment and stopping loratadine. After 4 months of maintenance, the family stopped the montelukast treatment by themselves. The patient also stopped food avoidance, and the free diet did not induce the recurrence of diarrhea.

The last follow-up was in December 2018; the patient had been discharged from the hospital for 11 months, and there was no recurrence of clinical symptoms. He continued the dust mite desensitization treatment. The total IgE levels had significantly reduced to 518.0 IU/ml, and ANA reduced to 1:100, AHA (–). His function of lung ventilation had essentially restored to normal. The patient's colonoscopy was re-checked on December 11, 2018, and showed completely normal on the mucosal image (see Fig. 1B1–B3). Pathological biopsy showed that invasion of the lymphocytes and plasma cells was evidently reduced compared with the previous time, and submucosal collagen bands were also significantly narrowed or had disappeared (see Fig. 1B4). CC in this patient was essentially cured both clinically, through the remission of diarrhea, and histologically. The general clinical information of the patient is summarized in Table 1.

**Table 1:**
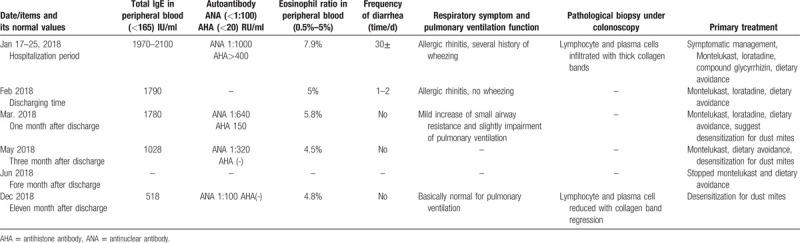
Summary for clinic and treatment of the patient.

## 3 Discussion

CC was first reported by Lindström in 1976,^[9]^ and is a type of MC. It is mainly characterized by chronic watery diarrhea, with or without abdominal pain. Appearances of the mucosa are almost always normal under colonoscopy, yet pathological biopsy can show specific signs under microscopy. Pathological biopsy is the direct diagnostic support for CC.^[5–7]^ Although the incidence of CC has increased in recent years,^[10]^ it is still a relatively rare disease,^[11–13]^ with the majority of previous reports on CC in adult women^[11,13]^; case reports dealing with child patients are extremely rare.^[2,14]^ Lymphocytic colitis (LC) is another type of MC that is characterized by extensive infiltration of the intraepithelial lymphocytes; yet CC shows as thickened collagen bands under the epithelia (more than 10 μm).^[15]^ Both LC and CC result in mononuclear infiltration of the lamina propria of the intestines.^[16]^ It is still controversial whether LC and CC are different diseases or different stages of the same disease.^[17–19]^ It has also been reported that CC and LC are two kinds of closely related, but independent, intestinal diseases.^[20]^ The patient in this report was a school-age boy who passed watery stools 30+ times per day for more than 1 month. His diarrhea symptoms were consistent with the clinical features of CC, and no mucosal abnormalities were found under colonoscopy (see Fig. 1A1–A3). However, pathological biopsy showed extensive collagen deposition and infiltration of the lymphocytes and plasma cells (see Fig. 1A4). The diagnosis was finally made clear through endoscopic biopsy. Clinical symptoms of CC are usually atypical, and endoscopic signs are not significant, which easily leads to misdiagnosis.^[21]^ This child had two colonoscopies in the primary hospital, yet all were misdiagnosed because no biopsy was conducted. Therefore, routine pathological biopsy is especially important for chronic diarrhea of unknown etiology. Even though no abnormality is shown under colonoscopy, biopsy is still a crucial way to assist diagnosis.

Moreover, it has been reported that one-third of MC are combined with immune-mediated diseases,^[4]^ such as thyroid disease, celiac disease, diabetes, psoriasis, and rheumatoid arthritis. In some cases, the diagnosis of autoimmune diseases preceded the diagnosis of MC.^[4]^ Several auto-antibodies can also be found in MC patients, such as rheumatoid factor, ANA, anti-mitochondrial antibodies, anti-neutrophil cytoplasmic antibodies, anti-saccharomyces cerevisiae antibodies, and anti-thyroid peroxidase antibodies.^[4]^ This indicates that MC is associated with immune diseases. As a type of MC, the pathogenesis of CC has not been completely clear until now. Several etiological factors related to CC in adults had been proposed, such as autoimmune diseases, genes, drugs, smoking, bile acid absorption disorders, and gastrointestinal flora dystopy.^[3]^ It was also reported that 17% to 40% of CC patients have autoimmune diseases, such as rheumatoid arthritis, collagen vascular disease, thyroid disease, and celiac disease.^[18]^ CC in children is very rare, and several cases have been related to celiac disease, Crohn's disease, juvenile scleroderma, eosinophilic gastrointestinal disease, aeromonas hydrophilia infection, and helicobacter pylori infection.^[14]^ The patient in this report also had increasing ANA and AHA auto-antibodies, which is consistent with the notion that CC is often induced by immune-allergic factors. However, in the early stages of the disease, it is usually difficult to determine if an immune-allergic reaction is the leading cause. Manifestations of CC in this child were mainly shown as gastrointestinal symptoms with no other systems involved. Therefore, all searches were focused on gastrointestinal infections and food allergies at the beginning, and no specific abnormalities were found. Soon after, significant increases in total IgE were observed.

Hyperimmunoglobulinemia E (hyper-IgE) is a clinical syndrome with complex causes, such as tuberculosis, fungus infection, hypersensitive reactions, parasitic infections,^[22]^ and atopic dermatitis.^[23]^ In our report, screening for specific pathogens such as parasites and tuberculosis was performed, and no evidence of infection was found in the patient. After screening for pathogens, immuno-allergic causes should be considered. Food allergy-specific IgE tests were all negative, and only several food antibodies of IgG were slightly elevated, but this finding was too weak to diagnose a food allergy. However, because of extremely severe diarrhea with poor effects, we also suggested that the patient avoid foods suspected of causing an allergic reaction. Unfortunately, the elimination diet did not improve his diarrhea. The patient still needed continued intravenous infusion to avoid dehydration. In order to trace the cause of hyper-IgE syndrome, we carefully inquired about the patient's medical history again and finally found that the child had mild allergic rhinitis and asthma. However, the patient had no respiratory symptoms during this course of diarrhea. Therefore, detection of respiratory specific IgE was performed, and multiple respiratory allergens were strong positives. This indicated that a respiratory allergy, rather than gastrointestinal allergy, may be the main cause of hyper-IgE syndrome. Anti-allergy treatments were given to the patient, according to this result. Diarrhea symptoms remarkably improved after routine anti-allergy treatment, which verified our speculation. Although diarrhea was completely relieved, the total IgE levels remained high, which suggested a high risk of recurrence. Corticosteroid treatment was recommended to the patient, combined with the final pathological diagnosis. However, his parents refused this treatment because of his diarrhea having relieved. According to the specific allergic reaction of this patient, desensitization to dust mites was recommended. After desensitization treatment for several months, the patient's symptoms continued to relieve. IgE levels, autoantibodies, and pathological biopsy were essentially restored to normal, which further confirmed that CC in this patient was induced by a respiratory allergy. The original cause of immuno-allergies was in the respiratory tract, not the gastrointestinal tract. This was a considerably particular CC case, which was originally induced by respiratory immuno-allergy. CC induced by respiratory allergy has not been previously reported in either adults or children.

Treatment of CC aims to achieve clinical remission (daily bowel movements <3 times or 1 watery stool).^[4,8]^ Histological remission is still controversial and difficult to achieve.^[4]^ Natural courses of MC and CC vary significantly, and the majority of patients have relatively benign courses. Some cases can develop into chronic intermittent or persistent diarrhea.^[4,12]^ Treatment of CC may be individualized, but searching and blocking inducement is critical for treatment, such as stopping non-steroidal anti-inflammatory drugs, quitting smoking,^[8]^ and lifestyle adjustments.^[3]^ Oral budesonide is currently the most effective treatment for MC/CC^[4]^ and is recommended for CC patients to help induce clinical remission.^[8,15,16]^ Numerous other treatments are also suggested, including anti-diarrhea drugs, bismuth subsalicylate, aminosalicylate, methotrexate, antibiotics, and infliximab.^[24]^ However, treating CC with anti-allergic drugs and desensitization therapy had not been previously reported. This patient was also a unique case who achieved complete remission both clinically and histologically.

Moreover, budesonide is an effective local corticosteroid, which can reduce the systemic side effects of steroid treatment.^[4]^ However, this patient was highly allergic to dust mites and had a history of allergic rhinitis and wheezing; therefore, the gastrointestinal and respiratory systems were both involved. If choosing oral budesonide, it will only affect the local tract of the gastrointestinal system and will not improve respiratory allergies. Therefore, systemic corticosteroids may have been more suitable for this patient than oral budesonide. We originally planned to recommend a short-term therapy of prednisone for the patient. However, his family refused steroid therapy because his diarrhea was relieved by anti-allergy medicine. Considering dust mites were his most significant allergen, we chose desensitization as an individualized treatment for this child. By progress of the desensitization treatment, the total IgE levels of the patient gradually decreased, the autoantibody turned negative, recurrence of diarrhea did not occur, and clinical symptoms were completely relieved. The pathology also significantly improved in the latest colonoscopy with biopsy, which proved that a respiratory allergy was the main pathogenesis of this CC case. By treating the respiratory allergy, not only were CC symptoms significantly improved, but histopathological remission was obtained, and corticosteroid treatment was deemed unnecessary for this patient. Therefore, pathogeny searching is considerably important for the treatment of CC. On the other hand, if the patient had accepted corticosteroid therapy, oral budesonide in the local gastroenterological tract would not have helped to treat his respiratory allergy; therefore, systemic steroid treatment may be preferable as a multiple system treatment.

## 4 Conclusion

For chronic diarrhea of unclear etiology, a routine biopsy is required, even if there are no abnormal signs under endoscopy. Moreover, etiology searching is a crucial step for CC treatment. Gastrointestinal allergy is a common cause of children's chronic diarrhea; however, in this case of CC, respiratory allergy was the cause of chronic diarrhea. This suggests that etiology exploration should not be limited to the digestive system, as multiple system searching is also necessary.

## Acknowledgments

We would like to give our thanks to the parents of the patient, who gave consent for the publication data and images of the patient under the situation of not disclosing the names and portrait of the family. We would also like to thank Dr. Bin Wei, Department of Pathology, West China Hospital of Sichuan University, who helped us immensely with the pathological diagnosis.

## Author contributions

**Conceptualization:** Xue-Meng Wan, Yong-Mei Xie.

**Data curation:** Li-Yuan Wang.

**Formal analysis:** Zhi-Ling Wang, Xiao-Tang Cai.

**Supervision:** Chao-Min Wan.

**Writing – original draft:** Xue-Meng Wan.

**Writing – review & editing:** Yong-Mei Xie.
